# Between-Monitor Differences in Step Counts Are Related to Body Size: Implications for Objective Physical Activity Measurement

**DOI:** 10.1371/journal.pone.0018942

**Published:** 2011-04-27

**Authors:** Jeremy Pomeroy, Søren Brage, Jeffrey M. Curtis, Pamela D. Swan, William C. Knowler, Paul W. Franks

**Affiliations:** 1 Diabetes Epidemiology and Clinical Research Section, National Institute of Diabetes and Digestive and Kidney Diseases (NIDDK), Phoenix, Arizona, United States of America; 2 Healthy Lifestyle Research Center, College of Nursing and Health Innovation, Arizona State University, Phoenix, Arizona, United States of America; 3 MRC Epidemiology Unit, Cambridge, United Kingdom; 4 Genetic and Molecular Epidemiology Unit, Department of Clinical Sciences, Lund University, Malmö, Sweden; Universidad Europea de Madrid, Spain

## Abstract

**Background:**

The quantification of the relationships between walking and health requires that walking is measured accurately. We correlated different measures of step accumulation to body size, overall physical activity level, and glucose regulation.

**Methods:**

Participants were 25 men and 25 women American Indians without diabetes (Age: 20-34 years) in Phoenix, Arizona, USA. We assessed steps/day during 7 days of free living, simultaneously with three different monitors (Accusplit-AX120, MTI-ActiGraph, and Dynastream-AMP). We assessed total physical activity during free-living with doubly labeled water combined with resting metabolic rate measured by expired gas indirect calorimetry. Glucose tolerance was determined during an oral glucose tolerance test.

**Findings:**

Based on observed counts in the laboratory, the AMP was the most accurate device, followed by the MTI and the AX120, respectively. The estimated energy cost of 1000 steps per day was lower in the AX120 than the MTI or AMP. The correlation between AX120-assessed steps/day and waist circumference was significantly higher than the correlation between AMP steps and waist circumference. The difference in steps per day between the AX120 and both the AMP and the MTI were significantly related to waist circumference.

**Interpretation:**

Between-monitor differences in step counts influence the observed relationship between walking and obesity-related traits.

## Introduction

The rising prevalence of diseases such as type 2 diabetes, obesity, heart disease and some cancers has been partially attributed to a decrease during recent decades in habitual physical activity [1], [2]. Walking is a common mode of physical activity [3], and is generally safe and requires little training, equipment or facilities. Thus, walking represents a logical target activity for health promotion.

Quantifying the relationships between walking and health-related traits requires accurate measurements of walking. Several devices are currently available for measuring steps taken, which vary greatly in complexity and cost. Most devices measure the total number of steps a person takes during the entire monitoring period, while some are also capable of storing time-stamped step counts, which enables analysis of intensity, duration, and frequency of walking bouts. The most common monitor for assessing daily step count is a mechanical pedometer [4], although accelerometers are also often used [5]. Step monitors can be used in research studies to motivate as well as to measure habitual physical activity. In several large clinical trials, including the Diabetes Prevention Program [6] and the Look AHEAD study [7], the relatively inexpensive hip-worn Accusplit AX120 spring-lever pedometer was used to motivate participants to walk more. A recent clinical trial used a pedometer intervention to encourage individuals with impaired glucose tolerance (IGT) to walk more [8]. In the DREW study [9] an Accusplit pedometer was used to measure unstructured physical activity in women randomized to exercise interventions that differed by time spent exercising. By contrast, the recent NHANES [10] used a comparatively expensive uniaxial accelerometer - the MTI ActiGraph, which in addition to measuring accelerometry counts is also capable of measuring steps (as movement frequency) to quantify physical activity. Another accelerometer-based step monitor is the AMP-331, which uses a pattern recognition algorithm applied to the triaxial acceleration waveform signals at the ankle to count steps taken during walking and running [11]. Some reports have suggested that during treadmill walking, error in spring-lever pedometers was greater in participants with higher waist circumference and BMI such that spring-lever pedometers might not capture as many steps taken as piezo-electric pedometers [12]. Little is known of how differences in the measurement of steps taken between monitors affect the interpretation of the relationships between walking and health outcomes.

The aim of this study was to examine how the relationship between steps per day and body size, glucose regulation, and adjusted physical activity level during free living differed between three different step counting monitors.

## Materials and Methods

Following a recruitment campaign in the Phoenix metropolitan area, 27 men and 27 women aged 20–34 years of ≥50% American Indian ancestry attended the National Institutes of Health Clinical Research Center (CRC) in Phoenix, AZ, at 7:00 am on two occasions separated by a 7-day period of free-living observation. Participants provided written informed consent and underwent a detailed medical screen to evaluate the risk of participation. The institutional review boards of the National Institute of Diabetes and Digestive and Kidney Diseases and the Phoenix Area Indian Health Service approved the protocol. No participants were taking medicines for treatment of hyperglycemia or high blood pressure.

### Clinical Measures

Participants arrived at the CRC after a 10-hr overnight fast. Blood was drawn before and 2 hours after a 75 g oral glucose challenge for assessment of glucose tolerance according to World Health Organization diagnostic criteria [13]. One enrolled volunteer (out of 54 total enrolled) had fasting and 2-hr blood glucose concentrations consistent with a diagnosis of diabetes and was thus excluded from further participation.

Standard anthropometric data were collected by trained observers with participants in a hospital gown and no shoes. Height and weight were measured using a rigid stadiometer and calibrated scale. The weight of the hospital gown was subtracted from each individual's body weight. Body composition was assessed using a calibrated fan beam dual-x-ray absorptiometer (DXA; Prodigy, GE/Lunar Co.).

### Energy expenditure

#### Resting energy expenditure

Resting energy expenditure (REE) was assessed shortly after arrival at the CRC on the final morning of measurement using a Parvo Medic TrueOne® 2400 (Parvo Medic, Salt Lake City, UT, USA) open-circuit metabolic cart configured for the assessment of resting respiratory gas concentrations using a ventilated hood. The machine was calibrated against gases of known composition (0.03% CO_2_, 20.94% O_2_, remainder N_2_) on the morning prior to each test by a trained experimenter. Participants were fitted with a metabolic hood while lying on a bed in a quiet room and were instructed not to sleep, speak or move during the test. The total measurement time was 45 minutes, but the first 7 minutes of data for all tests were excluded to allow for metabolic stabilization and the remaining test data were averaged for each individual.

#### TEE from doubly-labeled water

Total free-living energy expenditure was assessed with doubly-labeled water (DLW) using methods previously described [14]. Participants provided a baseline urine sample on admission to the clinic in the morning of the first visit. In the late morning of the same day, the participants voided and drank a 1.9 g dose of DLW (0.0896 g ^2^H_2_O and 0.181 g H_2_^18^O in a 1:20 ratio of ^2^H: ^18^O per kg of total body water as estimated from body weight and height). Repeat urine samples were then collected 2 hr, 3.5 hr, and 5 hr after dosing while the participant remained at the CRC. Seven days later, the participant returned to the clinic in the morning after an overnight fast and provided two additional timed urine samples collected during the ensuing 4 hrs. The ^18^O and ^2^H isotopic enrichments in the urine samples were measured shortly after collection. Mean daily CO_2_ production (rCO_2_ in moles/day) was calculated, from which total daily energy expenditure (TEE) was derived assuming a food quotient of 0.87.

#### Physical Activity Level and Physical Activity Energy Expenditure

Physical activity energy expenditure (PAEE) in kilocalories/day was calculated as (0.9*TEE – REE). Adjusted physical activity level (adjusted PAL) was calculated as the residual of a regression predicting TEE from REE [15]. The purpose of the adjusted PAL is to make physical activity comparisons between individuals of differing body size, but avoid the drawbacks inherent in the use of ratios [16].

### Physical activity monitors

Three different monitors, all capable of measuring steps, were used for both laboratory and free-living conditions; the hip-worn Accusplit AX120 spring-lever pedometer (San Jose, California, USA), the hip-worn MTI ActiGraph model 7164 uniaxial accelerometer (Fort Walton Beach, Florida, USA), and the ankle-worn Dynastream AMP-331 triaxial accelerometer (Cochrane, Alberta, Canada). The step counting feature of the MTI (or its predecessor the CSA) has been used as a criterion measure to determine pedometer accuracy in free-living conditions [17]. The AMP is specifically designed to assess additional dimensions of locomotion, including speed and stride length. While the MTI and AMP have additional capabilities, in this investigation, only the total steps measures were compared.

### Assessment of monitor step count accuracy (laboratory)

The participants underwent a walk test. Each participant was fitted with the monitors and instructed to walk at a self-selected pace around a 6-lap level and even course on the hospital floor (540 meters total distance). The AX120 and MTI were both attached to a neoprene belt, the MTI was positioned at the level of mid-axillary and the AX120 was positioned at the level of the mid-thigh. The AMP was placed in a manufacturer provided sleeve that was worn around the ankle over the participant's sock. During the test, a trained observer ensured that the participant neither deviated from the course nor was in any way prevented from walking at his or her chosen pace. The observer recorded the number of steps taken using a thumb-click unit counter. The absolute discrepancy for each monitor was determined as the median absolute value of ((steps detected – observed steps)/observed steps) ×100%.

We calculated a signed discrepancy score for each monitor to assess the relationships between monitor step counts and observed step counts, as well as step counts between the monitors. We also calculated signed discrepancy scores to assess how measurement in the laboratory setting compared with measurement in free living.

To compare measurement discrepancies between the monitors in the laboratory versus free living we calculated discrepancy scores that did not use observed steps. The laboratory discrepancy scores were calculated as ((AX120 step counts – AMP step counts)/AMP step counts); ((MTI step counts – AMP step counts)/AMP step counts); and ((AX120 step counts – MTI step counts)/MTI step counts). The free-living discrepancy scores were calculated as ((AX120 steps/day – AMP steps/day)/AMP steps/day); ((MTI steps/day – AMP steps/day)/AMP steps/day); and ((AX120 steps/day – MTI steps/day)/MTI steps/day).

### Assessment of steps taken during free-living

Steps/day were measured with the AX120, MTI, and AMP during a 7-day free living test period. The participants were instructed to wear the monitors in the same configuration as during the walk test. Participants were advised not to remove the AX120 or MTI from the belt and to remove the belt and AMP for sleep and periods of water immersion. For this investigation, we assumed that the AX120 was worn (or not worn) simultaneously with the other monitors. We used previously published methods to discern non-wear periods in the MTI [18]. Briefly, this method uses periods of near-continuous zero counts lasting at least 60 minutes to discern non-wear. We also examined the time-stamped output of the AMP to verify that no steps were counted during periods of MTI non-wear. We did not include any days with less than 10 hours of wear (according to the MTI) in the analysis. We assumed that non-wear time was spent in sedentary behaviour (not in locomotion). Participants were given a diary with space to record details about monitor wear and removal during the free-living period. Participants were asked to indicate time and reason for device removal in the provided activity diary. According to the diaries kept by volunteers, only one participant engaged in an activity in which the monitors could not be worn. This participant reported one episode of swimming during the study period, described as “lounging at a pool” (i.e. not swimming for exercise).

### Statistical analysis

Spearman correlations, partialled for age and sex, were used to test the associations between steps counted by each monitor and body size, glucose regulation, adjusted PAL, and PAEE. We also tested the equality of the correlations between monitors and each of the body size, glucose regulation, and physical activity measures [19]. The slope between PAEE and 1000 steps per day was calculated for each monitor using generalized linear models. Statistical significance was defined as a p-value ≤0.05 for all statistical tests. All statistical analyses were performed using SAS version 9.2 (SAS Institute, Cary, NC).

## Results

Complete data were available for 50 participants (25 women), characteristics of whom are shown in **Table 1**. One participant was unable to complete the walk test, data could not be retrieved from one AMP monitor and one MTI monitor. Of the men, 48% were obese (BMI ≥30 kg/m^2^). Of the women, 20% were obese. The mean (range) wear time for the MTI was 14.1 (11.4–16.0) hours per day for women and 14.7 (12.3–18.4) hours per day for men.

**Table 1:** Descriptive characteristics of participants.

Variable	Men	Women
N	25	25
Age (years)	26.8 (24.0, 31.1)	24.9 (23.0, 29.7)
Height (cm)	178.7 (175.0, 183.1)	163.4 (161.4, 167.6)
Weight (kg)	94.6 (82.6, 117.5)	68.7 (63.1, 73.4)
Waist circumference (cm)	105.0 (92.0, 120.7)	85.4 (80.0, 92.0)
BMI (kg/m^2^)	30.0 (26.4, 36.6)	25.6 (23.4, 27.8)
Body fat (%)	35.5 (29.1, 42.3)	35.4 (31.9, 41.4)
Total energy expenditure (kcal/d)	3580 (3453, 3825)	2392 (2088, 2560)
Resting energy expenditure (kcal/d)	1890 (1673, 2108)	1288 (1200, 1458)
Physical activity energy expenditure (kcal/d)	1358 (1120, 1482)	764 (687, 930)
Walking speed during walk test (km/h)	4.74 (4.37, 5.12)	4.82 (4.54, 5.40)
Steps/day from AX120 (hip-worn pedometer)	5668 (3865, 7988)	5516 (4178, 7876)
Steps/day from MTI (hip-worn accelerometer)	10064 (7671, 12536)	8824 (7282, 10467)
Steps/day from AMP (ankle-worn accelerometer)	8585 (5761, 11029)	6865 (6115, 8311)

Statistics are medians (25^th^, 75^th^ percentile).

The median (25^th^, 75^th^ centiles) step count discrepancies between monitors are shown in **Table 2**. The 3–6 fold differences between laboratory comparisons and free-living comparisons indicate that step count differences are much greater in the free-living environment than in the controlled laboratory walk test.

**Table 2:** Discrepancy scores for three different objective methods for assessing steps.

	Men		Women	
N	25		25	
	AbsoluteDiscrepancy	SignedDiscrepancy	AbsoluteDiscrepancy	SignedDiscrepancy
540m laboratory walk test				
AMP steps versus observed steps	0.5 (0.1, 1.1)	-0.1 (-0.8, 0.5)	0.3 (0.0, 0.9)	0.0 (-0.4, 0.5)
AX120 steps versus observed steps	3.6 (1.3, 11.7)	0.1 (-5.6, 1.6)	4.4 (0.5, 10.8)	-1.6 (-8.8, 0.1)
MTI steps versus observed steps	5.6 (2.6, 9.8)	-0.1 (-5.6, 5.5)	7.5 (3.2, 11.4)	2.8 (-3.8, 8.4)
AX120 walk versus AMP steps	4.4 (1.6, 11.4)	-1.8 (-6.4, 1.3)	3.7 (0.8, 9.5)	-0.6 (-8.4, 0.9)
MTI steps versus AMP steps	4.9 (3.0, 9.0)	0.0 (-4.9, 4.7)	6.2 (3.3, 11.1)	4.2 (-2.1, 8.7)
AX120 steps versus MTI steps	6.0 (2.0, 17.8)	-2.0 (-10.5, 1.1)	10.4 (7.1, 16.1)	-7.7 (-16.6, -1.2)
Free-living				
AX120 steps/day versus AMP steps/day	26.6 (12.0, 41.5)	-26.6 (-41.5, -12.0)	22.5 (11.3, 28.5)	-22.0 (-28.5, -9.6)
MTI steps/day versus AMP steps/day	17.4 (8.8, 30.6)	-16.4 (-22.9, -7.6)	18.0 (13.9, 28.0)	-18.7 (-29.9, -13.5)
AX120 steps/day versus MTI steps/day	20.2 (7.6, 32.1)	-11.4 (-30.4, 2.2)	15.2 (8.6, 26.3)	7.9 (-9.1, 15.7)

Statistics are medians (25^th^, 75^th^ percentile) percent difference.

**Figure 1:**
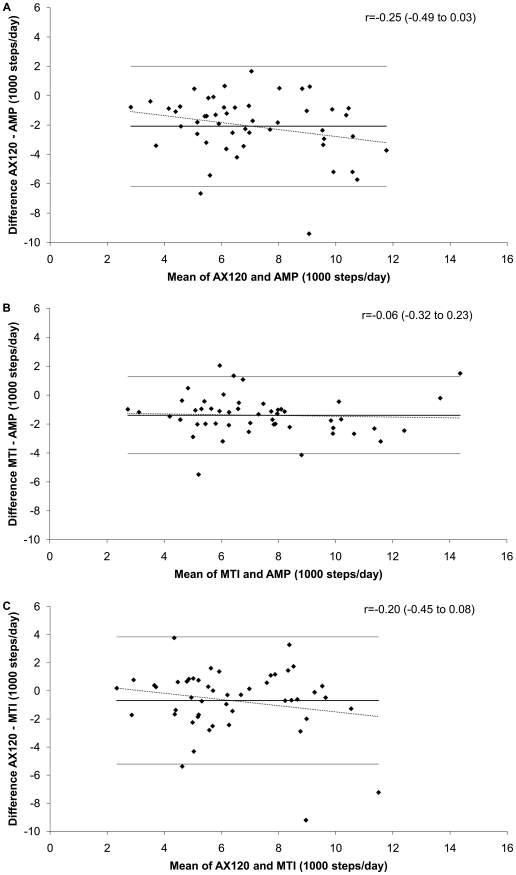
Bland-Altman plots of agreement between free-living steps per day. The pairwise comparisons of the difference between steps per day (shown in 1000 steps per day) plotted by the average steps per day. The correlation coefficients and 95% confidence intervals are for the relationship between the difference in steps per day between the monitors and the average steps per day of the monitors. A significant correlation indicates significant heteroscedasticity. Figure 1A shows the difference between the AX120 hip-worn pedometer and the AMP-331 ankle-worn accelerometer by the average of the AX120 and AMP steps per day. Figure 1B shows the difference between MTI hip-worn accelerometer and AMP-331 ankle-worn accelerometer steps per day by the average of MTI and AMP steps per day. Figure 1C shows the difference between MTI hip-worn accelerometer and AX120 hip-worn pedometer steps per day by the average of MTI and AX120 steps per day.

**Figure 1** shows the Bland-Altman plots of the pairwise comparisons of the difference in steps/day measured by each of the monitors plotted by the average steps/day. The AX120 generally undercounted relative to either of the other step counters. The difference in steps/day was not significantly related to the average steps/day for any of the pair wise comparisons.

**Figure 2:**
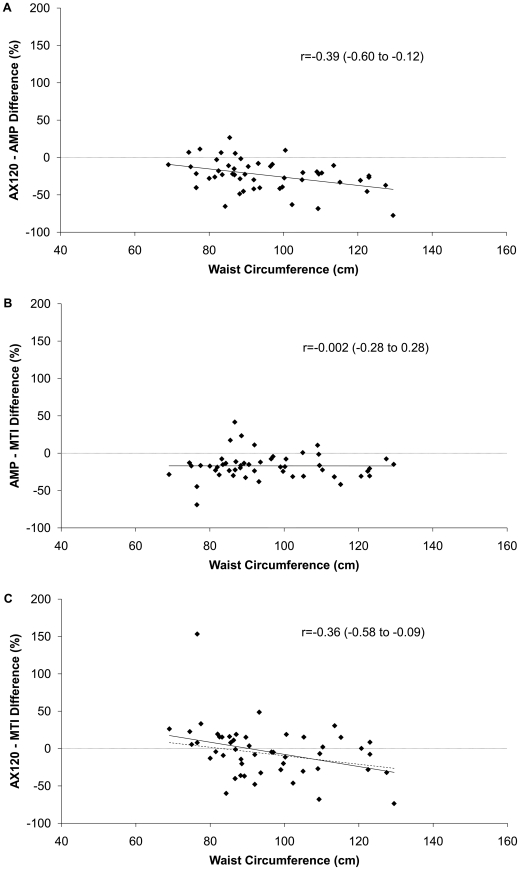
Relationship between differences in steps per day and waist circumference. The difference in steps per day, expressed as a percent difference, is plotted by waist circumference. The correlation coefficients and 95% confidence intervals are for the relationships between percent difference in steps per day and waist circumference. A significant correlation indicates a relationship between percent difference in steps per day and waist circumference. Figure 2A shows the relationship between the percent difference in AX120 hip-worn pedometer and the AMP-331 ankle-worn accelerometer (expressed as a percentage of the AMP-331) steps per day and waist circumference. Figure 2B shows the relationship between the percent difference in AMP-331 ankle-worn accelerometer and MTI hip-worn accelerometer (expressed as a percentage of the MTI) steps per day and waist circumference. Figure 2C shows the relationship between the percent difference in AX120 hip-worn pedometer and the MTI hip-worn accelerometer (expressed as a percentage of the MTI) steps per day and waist circumference.

**Figure 2** shows the correlations between the pairwise percent difference in steps/day during the free living trial and waist circumference. The difference in steps/day measured by the AX120 was significantly negatively related to waist circumference when compared to either of the other monitors.

**Table 3** shows the Spearman correlation coefficients for free-living steps/day and the metabolic health parameters. In general, AX120 and MTI steps/day were inversely related to indicators of obesity, whereas corresponding correlations with AMP steps/day were weaker and not statistically significant. Fasting glucose was inversely related to all step measures but differences between correlations did not reach statistical significance.

**Table 3:** Associations between steps taken and metabolic health outcomes.

Measure	AX120 (hip-worn pedometer) steps/day	MTI (hip-worn accelerometer) steps/day	AMP (ankle-worn accelerometer) steps/day
	r (95% CI)	r (95% CI)	r (95% CI)
BMI	-0.38 (-0.59 to -0.11)	-0.25 (-0.50 to 0.04)	-0.21 (-0.46 to 0.08) a
Weight	-0.41 (-0.62 to -0.15)	-0.33 (-0.56 to -0.04)	-0.24 (-0.49 to 0.05) a
Waist circumference	-0.43 (-0.63 to -0.15)	-0.26 (-0.51 to 0.03)	-0.18 (-0.44 to 0.10) a
Fat mass	-0.41 (0.62 to -0.15)	-0.27 (-0.52 to -0.02)	-0.17 (-0.43 to 0.12) a
Fat-free mass	-0.25 (-0.50 to 0.05)	-0.24 (-0.49 to 0.04)	-0.22 (-0.47 to 0.06)
Fasting glucose	-0.44 (-0.64 to -0.17)	-0.40 (-0.61 to -0.12)	-0.30 (-0.54 to -0.01)
Two hour glucose	-0.23 (-0.49 to 0.06)	-0.09 (-0.37 to 0.20)	-0.16 (-0.43 to 0.13)
Fasting insulin	-0.18 (-0.46 to 0.12)	-0.17 (-0.44 to 0.14)	-0.17 (-0.44 to 0.14)
Adjusted PAL	0.34 (0.05 to 0.57)	0.44 (0.17 to 0.64)	0.43 (0.16 to 0.63)
PAEE	0.18 (-0.10 to 0.43)	0.47 (0.21 to 0.66) b	0.42 (0.15 to 0.62)^a^

Spearman correlations between steps/day and BMI are partialled for age and sex, all other correlations are additionally partialled for height.

a p≤0.05 for difference between AMP and AX120.

b p≤0.05 for difference between AX120 and MTI.

Steps/day as measured by MTI or AMP were both significantly positively related to physical activity measures from doubly-labeled water, and more strongly so than the AX120. **Table 4** shows the results of simple linear regressions predicting PAEE from steps counts from each of the monitors. Both the AMP and the MTI step counts were significant correlates of PAEE while AX120 step counts were not.

**Table 4:** Simple linear regressions predicting PAEE (kcal/d) from step counts (expressed per 1000 steps/day).

Predictor	Intercept	β coefficient (change in kcal/d per change in 1000 steps/day)	95% Confidence Interval for β coefficient
AMP	2422	262	117 to 409
MTI	2628	286	139 to 433
AX120	3754	131	-65 to 328

## Discussion

Pedometers and other types of step counters have been used widely to measure physical activity [9], [10], [20], [21] but vary considerably in accuracy, with the AMP accelerometer and the MTI accelerometer thought to be among the most accurate monitors for step counting [17], [22]. Accuracy is often assessed during short walking tests done on treadmills or other lab-based settings. In these lab-based studies error in spring-lever devices has been shown to be positively associated with BMI, walking speed, and pedometer tilt [12], [23], [24]. It is plausible that due to placement, an ankle-worn accelerometer (e.g., the AMP) would not be as prone to error related to body size as a hip-worn spring-lever pedometer (e.g., the AX120). Indeed, the AMP was the most accurate of the instruments compared with observed steps in the laboratory-based walk test. A different ankle-worn step counting device (Stepwatch) was more accurate than hip-worn devices adults [22] and in normal and overweight children at walking speeds greater than 0.3 kph [23] in laboratory-based tests. In our investigation the step counting discrepancies were considerably larger in the 7-day free living trial compared with the 540m walking test in the clinical research center. This highlights the difficulty in using laboratory-based tests to draw conclusions about step counter accuracy in free living.

Comparison of devices in free living are often only conducted over a 24 hour measurement period [12], [17], [22]. These studies consistently find that spring-lever pedometers are less accurate than either piezo-electric pedometers or accelerometry-based devices. In a recently published paper, error for a spring-lever pedometer (similar to the AX120 we used), defined as discrepancy from steps counted by an ankle-worn device, was positively related to BMI, such that the spring-lever device undercounted steps to a greater extent in obese individuals during 7 days of free-living [26]. This is consistent with our finding. The differences between AX120 steps/day and AMP steps/day as well as AX120 steps/day and MTI steps/day were significantly related to waist circumference. Our study included individuals with a wide range of body sizes. The relationship between undercounting by the AX120 and waist circumference would be more difficult to detect in a sample that only included individuals with a more narrow range of body sizes, particularly if all the participants were of normal BMI and waist circumference. It is unclear why the AX120 undercounted relative to the other devices to a greater extent in individuals with a greater waist circumference. Free-living walk speed (as estimated by the AMP) was not significantly related to waist circumference in our study (data not shown). It is possible that other differences in walking biomechanics, patterns of walking, or the impact of waist circumference on the tilt of the device might influence steps captured by the AX120.

We show here that estimates of daily step accumulation vary considerably between monitors worn simultaneously during free living. This suggests that applying a determination about physical activity level using generic steps per day thresholds can result in different activity classifications based on the device used. A better approach to activity classification by steps per day would be to identify steps per day thresholds that are unique to the monitor used.

The correlations between the steps/day and measures of obesity were significantly different when using the AMP versus the AX120. The correlations between steps/day and PAEE were significantly different for the AMP versus the AX120 as well as between the AX120 and the MTI. Additionally, while the MTI and AMP step counts were each significant correlates of PAEE, the AX120 step counts were not. This suggests that it would be harder to detect changes in energy expenditure based on changes in steps per day using the AX120. This is relevant if the AX120 was to be used to set physical activity goals to increase daily physical activity energy expenditure. Daily step accumulation has been related with various measures of obesity [20], [21], [27], [28] and walking interventions are associated with favorable changes in cardiovascular risk factor profiles [29]. As shown in short walking tests, the Accusplit AX120 and other pedometers are susceptible to measurement error [17], [30], [25], [24]. Our results suggest that these correlations may be specific to the monitor used and might not be replicated with monitors that are believed to be more accurate. Our results also suggest that measurement accuracy impacts on the observed dose-response relationships between steps per day and health outcomes; this is somewhat concerning if an inaccurate measure was used to establish public health recommendations.

In the Diabetes Prevention Program and other clinical trials, pedometers were predominantly used to motivate participants. Recent research has suggested that the addition of a pedometer to a walking intervention was associated with a significant improvement in glucose tolerance compared to an intervention without using pedometers in individuals with IGT [8]. It is unclear if the accuracy of the step counting device used to motivate participants has an impact on intervention efficacy. We are unaware of any study assessing the impact of the accuracy of feedback of a physical activity monitor on intervention efficacy. Given the important ramifications for using step counting devices to motivate increased physical activity behavior we feel that this is an area that deserves future study. Devices such as the AMP and MTI may not be useful devices to motivate participants, because neither provides user feedback in the form of a real time display of steps taken. Spring-levered pedometers (such as the AX120) cost much less than accelerometers (such as the MTI and AMP). All of these factors must be taken into account when choosing a step monitor.

In conclusion, randomized clinical trials have shown the efficacy of lifestyle modification to prevent or delay the onset of type 2 diabetes in high-risk populations [2], [31]. Although these studies show convincingly that lifestyle modification decreases diabetes risk, little is known of the mechanisms that underlie this effect. To elucidate these mechanisms will likely require the application of accurate and precise measures of physical activity to appropriately designed studies. Therefore, the magnitude of the measurement error and the types of bias that are characteristic of different physical activity monitors should be considered when planning studies and interpreting data on the relationship between physical activity and health outcomes.
